# Wheatstone bridge configuration for evaluation of plasmonic energy transfer

**DOI:** 10.1038/srep24423

**Published:** 2016-04-14

**Authors:** J. Gosciniak, M. Mooney, M. Gubbins, B. Corbett

**Affiliations:** 1 Tyndall National Institute, University College Cork, Lee Maltings, Prospect Row, Cork, Ireland; 2 Seagate Technology, 1 Disc Drive, Springtown Industrial Estate, Londonderry, Northern Ireland, BT48 OBF.

## Abstract

We propose an internal (on-chip) Wheatstone bridge configuration to evaluate the efficiency of near-field transducers (NFT) as used in heat-assisted magnetic recording (HAMR). The electric field enhancement between the transducer and the image plane is monitored by measuring the resistance of metal electrodes composing the image plane. The absorption of the enhanced electric field causes an increase in the metal temperature, and thereby, in its resistance whose variation is monitored with an internal Wheatstone bridge which is accurately balanced in the absence of the electric field.

The rapid progress in plasmonics comes from its extraordinary properties which are not achievable with conventional photonics technology^1^,^2^,^3^. Plasmonic effects arise from the interaction of light with the cloud of free electrons in the metal which support, as a response, waves of charge density fluctuations on the surface of the metal. These enhanced free electron oscillations can exist as a localized surface plasmon resonance (LSPR)^1^ with the local electromagnetic field intensity enhanced by many orders of magnitude or as a propagating surface plasmon polariton (SPP)^1^ with light confined to an area much smaller than that predicted by the diffraction limit. These properties are of special importance because they open a horizon of new physical phenomena with applications enabling realization of nanoscale chip level interconnects^4^,^5^, ultra-small optical nanoantennas^6^,^7^, detectors^8^ and sensors^9^.

In the last decade the functionality of plasmonics has been extended to data storage applications where plasmonic antennas are able to shrink the optical focused spot far below the diffraction limit of conventional optics down to sizes of tens of nanometers^10^,^11^. This property enables a new technique called heat-assisted magnetic recording (HAMR) to overcome existing limits of conventional magnetic recording with an areal density approaching 1 Tb/in^2 ^12. HAMR exploits a near field transducer (NFT) to act as an antenna to locally heat the recording media in order to lower the coercivity of very high anisotropy materials to maintain data thermal stability and the ability to record and retain information at nanometre dimensions. The NFT design is based on the excitation of surface plasmons on a metal structure, which re-radiate a sub-diffraction limited light spot confined in the near field^12^,^13^,^14^. To satisfy requirements of a commercial technology, a few milliwatts of light power must be delivered to a 20 nm × 20 nm spot on the recording media to heat the media by 400 K to the Curie point in less than 1 ns which accounts for 1% of the laser output power^10^,^11^,^12^. It has recently been shown that one of the obstacles to the efficient coupling of power to the recording media is related to the inherent impedance mismatch between the media impedance and the radiation resistance of the transducer. This impedance mismatch can be reduced by the use of a larger transducer which can support a useful higher order mode^15^. Furthermore, the system efficiency can be further increased by integrating the transducer with a Mach-Zehnder Interferometer (MZI) planar waveguide configuration to match, in a most efficient way, the illumination from the waveguide to the radiation pattern of the antenna^16^.

While the true evaluation of a particular NFT configuration is in a functioning write head, it is necessary to evaluate and optimise different transducer configurations. Among many methods to characterize the efficiency of transducers such as dark-field microscopy^17^, second-order two-photon photoluminescence^18^, the scattering-type scanning near-field optical microscopy (s-SNOM) proves to be an essential tool capable of imaging plasmonic structures with a sub–10 nanometer resolution^19^. It can provide numerous valuable data about the transducer performance such as the field enhancement and the spot size at the recording media that are of special importance for data storage applications. However, it does not allow an estimate of the temperature increase that would be expected in the recording media which depends on both the field enhancement and impedance of the transducer and.

In this paper, we propose a novel measurement setup based on the Wheatstone bridge configuration that allows both very precise evaluation of the transducer and the expected temperature rise in the recording media with very high accuracy below ~0.1 K^20^.

## Results

**Figure 1:**
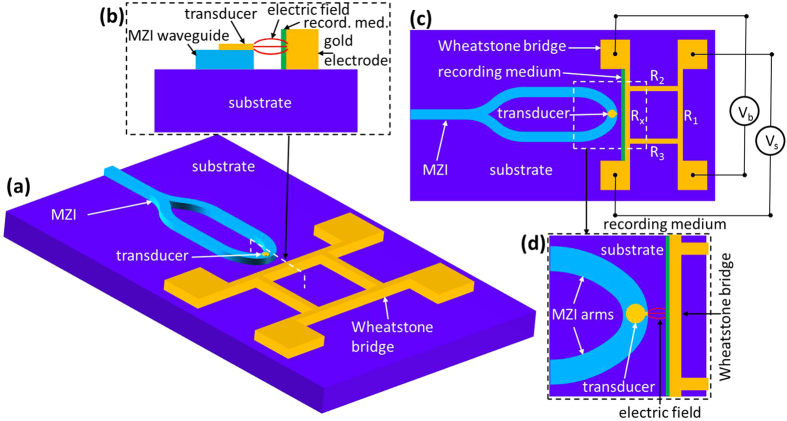
(**a**) Schematic layout of the Mach-Zehnder interferometer planar waveguide arrangement for coupling light to a near-field transducer with the Wheatstone bridge configuration and (**c**) top view of the structure. (**b**) Cross-section of the structure with the electric field enhanced between the transducer and image plane being at the same time part of the Wheatstone bridge structure. (**d**) top view of the transducer to recording media with gold electrode/image plane.

The proposed configuration for evaluation of the HAMR head and for monitoring the temperature rise in the recording media is based on a previously reported arrangement^5^,^20^,^21^ where the inevitable power losses in plasmonic waveguides were turned into a useful functionality by mode power monitoring. Here, the electric field enhanced in the gap between the transducer and its image plane, which simultaneously serves as one of the resistors in the Wheatstone bridge configuration, can be monitored by measuring the variation in the resistance of the metal stripe serving as an image plane caused by heating due to the mode absorption (Fig. 1). The enhanced electric field between the transducer and the metal stripe image plane, results in power transfer to the recording media and consequently a temperature rise. The optical power absorbed by the recording media is dissipated by the metal stripe. The dynamic increase of the image plane temperature Δ*T(t)* due to the absorption of the localized surface plasmon (LSP) mode power at time t can be expressed as


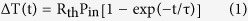


where *R*_*th*_ is the thermal resistance of the recording media, *P*_*in*_ is the power absorbed by the recording media and *τ* is the thermal time constant needed to load thermally the recording media. The total power absorbed in the recording media can be calculated using the complex Poynting vector theorem and Maxwell’s equations with the Poynting vector given by


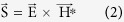


Thus, for the lossy medium, the power density dissipated can be expressed as


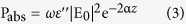


with the absorption coefficient *α~ε″/*(*ε′* )^2^. As a consequence, the power absorbed by the recording media depends strongly on its permittivity and the electric field E_0_ as


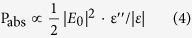


**Figure 2:**
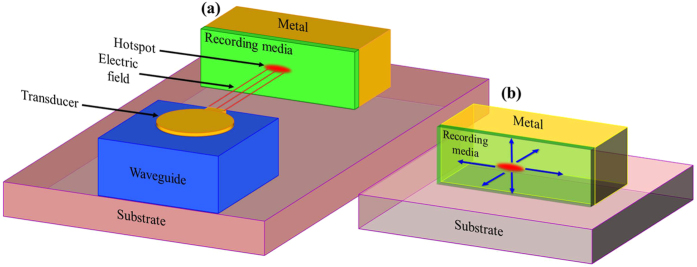
(**a**) An optical system with the NFT on the top of the waveguide (blue) to couple a light to the recording media (green) on the side of the metal electrode (yellow) and supported by the substrate. (**b**) Detailed view on the recording media with the lines (blue) showing the main direction of possible heat dissipation.

The imaginary part of the material permittivity, *ε″* determines the absorbed power. However, the real part of the permittivity plays an important role as it can affect the absorbed power through its impact on the electric field inside the recording material. The temperature rise causes an increase in the metal resistivity and, consequently, in the stripe resistance as follows


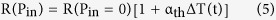


where *α*_*th*_ is the thermal resistance coefficient of the metal (*α*_*th*_ = 3.715·10^−3^ [1/K] for gold). As can be noted, the resistance increases linearly with the coupled power and, thereby, with the field enhancement. Thus measuring the metal stripe resistance enables monitoring of the power transfer to the recording media (Fig. 2). In the absence of the electric field enhancement between thea transducer and the image plane the signal voltage is given by


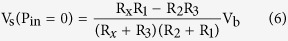


implying that for a perfectly balanced bridge, (*V*_*s*_ = 0), then *R*_*x*_*R*_*1*_ = *  R*_*2*_*R*_*3*_. Furthermore, to ensure the maximum response of the Wheatstone bridge all of the resistors should be designed to be equal in the absence of incident power *(P*_*in*_ = 0), i.e., *R*_*1* = _* R*_*2* = _* R*_*3 = *_* R*_*x*_, in which case the signal voltage can be expressed in terms of the temperature increase as


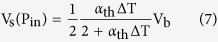


where *V*_*b*_ is the bias voltage.

The performance of different transducers can be easily evaluated in terms of their responsivity, i.e., power dependent signal voltage, by first measuring the signal voltage *V*_*s*_ of the Wheatstone bridge in the absence of the field enhancement (*P*_*in*_ = 0) and then measuring the signal voltage for different laser powers. Furthermore, the presented configuration enables determination of the temperature rise of the recording media through eq. 4.

**Figure 3:**
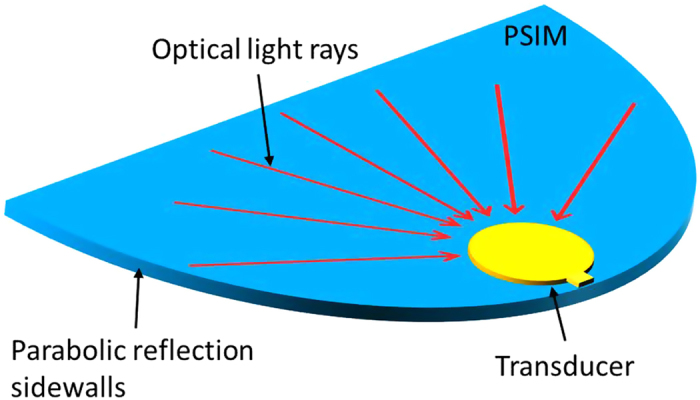
A planar solid immersion mirror (PSIM) and near-field transducer (NFT) placed at the focus of a waveguide with beams coupled to the NFT with different angles.

**Figure 4:**
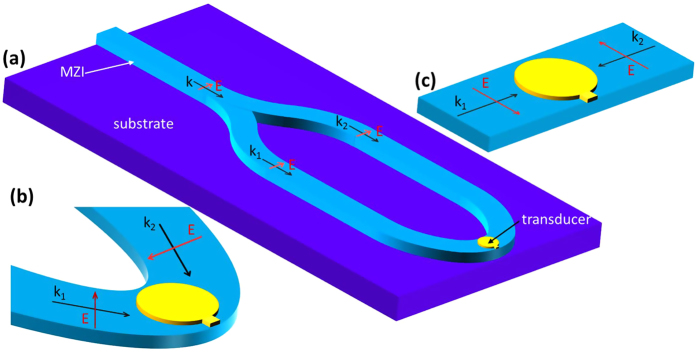
(**a**) Mach-Zehnder interferometer planar waveguide coupling arrangement for coupling light to a NFT located at the output Y junction. (**b**) Light coupled to a transducer with flexible coupling angle with two beams coupled to a transducer with a π-phase shift and (**c**) with a 180° coupling arrangement.

In addition, the configuration gives the possibility to evaluate the performance of both the transducer and coupling arrangement in terms of the temperature increase in the recording media by calibration of the system. For the same coupling configuration and with the recording media replaced by a polymer of a known melting temperature, the power required to melt a polymer can be used to evaluate the performance of different transducers under the same coupling arrangements. Furthermore, in the same way the efficiency of different coupling arrangements such as Planar Solid Immersion Mirror (PSIM) (Fig. 3) or MZI (Fig. 4), can be easily evaluated.

### Power transfer to metal electrode

The role of the NFT in the HAMR system is to increase the temperature of the recording media in a spot size as small as 20 nm × 20 nm. The enhanced electric field between the NFT and image plane transfers power to the recording media and heats it through the Joule heating mechanism. The power is then dissipated mostly into the metal on which the recording media is deposited and which acts as an image plane. It should be noted that the image plane here is also one of the electrodes of the Wheatstone bridge (Fig. 2a). Two main processes responsible for the temperature increase in the recording media can be distinguished. The first is related with radiative heat transfer from the NFT to the recording media which is very weak for a NFT composed of gold. The second is related with the field enhancement in the gap is the main heat transfer mechanism (eqs 3 and 4). Furthermore, it should be remembered that power absorbed by the recording media is transferred into heat that is then dissipated to any material that is in contact with it (Fig. 2b). However, the amount of heat transferred to any material depends on its thermal conductivity, contact area and heat dissipation length. For a practical device we take gold as the metal electrode, FePt^12^,^22^,^23^,^24^ as the recording media and SiO_2_ as the substrate material supporting the recording material and the gold electrode. We assume that the remaining sides of the recording layer and electrode are in contact with air. For such an arrangement, conductive heat transfer is assumed within solid and between solid object constituents, while convective heat transfer is considered between the surfaces of the materials in contact with air.

For such an arrangement, the amount of heat transferred to the metal electrode depends upon the thermal conductivity coefficients of the recording media (FePt-*κ* = 5 (W/m·K)–lateral direction, and *κ* = 50 (W/m·K)–vertical direction^23^), materials below the electrode (SiO_2_-*κ* = 1.4 (W/m·K)), along with the contact area and the thickness of the recording media. Furthermore, due to high thermal conductivity coefficient of gold (Au-*κ* = 100 (W/m·K)^23^) it can be treated as a heat sink and a convection coefficient of 10 (W/m^2^·K) was assumed for convective cooling of the materials in contact with air. Assuming a thickness for the recording media of *L* = 5–10 nm and a contact area A between the recording media and both gold and air defined by the width and height of the recording media (for example, *w* = 10 μm, *h* = 0.5 μm) it can be clearly concluded that due to:

- higher contact area of the recording media with gold electrode compared to the SiO_2_ substrate (100 times)
- much shorter heat dissipation length towards the gold electrode (50 times shorter)
- 10 times higher thermal conductivity coefficient of FePt in the vertical direction (towards gold electrode) compared to its lateral direction (towards substrate and air)
- much higher thermal conductivity coefficient of gold compared to SiO_2_ substrate (220 times higher) and air (300 times higher)

the lateral thermal diffusion is suppressed and the heat flows only in one direction namely towards the gold electrode/image plane. Thus, almost all the power absorbed by the recording media is dissipated into the gold electrode which gives rise to its temperature increase that is described by eq. 1. Thus, assuming that *P*_*in*_ = 1 mW of light power is delivered to A = 20 nm × 20 nm spot on the FePt recording media that is *L* = 10 nm thick, and for steady-state conditions, where Δ*T = R*_*th*_*·P*_*in*_ (eq. 1), with the thermal resistance defined as *R*_*th*_* = L/(κ·A),* we can obtain the *R*_*th*_ = 5·10^5^ (K/W), and consequently, a temperature rise of the recording media of Δ*T* = 500 K. Consequently, by assuming that 50% of power absorbed by the recording media is dissipated in the metal stripe, image plane having a cross-section of 500 nm × 500 nm and length of 10 μm and assuming a gold metal stripe with thermal conductivity coefficient of *κ* = 100 (W/m·K)^23^ we obtain a gold thermal resistance of *R*_*th*_ = 4·10^5^ (K/W) and stripe temperature rise of Δ*T* = 200 K (eq. 1). In the absence of the enhanced electric field between the transducer and image plane the gold stripe resistance is calculated at *R* ≈ 0.98 Ω (eq. 5) which rises to *R* ≈ 1.70 Ω (eq. 5) for a stripe temperature rise of Δ*T* = 200 K. Such a temperature rise will produce a signal voltage of *V*_*S*_ ≈ 27 mV for a bias voltage of *V*_*b*_ = 200 mV (eq. 7).

### Wheatstone bridge configuration for HAMR evaluations

#### Different coupling arrangements

One of the possible applications of the proposed Wheatstone bridge configuration measurement technique is an evaluation of the HAMR head in terms of the coupling efficiency of light to the NFT. In this case, the coupling system can be evaluated in terms of the signal voltage increase for the same power delivered from the laser to the transducer.

The significance of the proposed measurement technique will be shown by a theoretical analysis of two different coupling arrangements. The first one is based on a planar solid immersion mirror (PSIM)^25^, and the second one is based on the newly proposed Mach-Zehnder interferometer coupling arrangement^16^. The PSIM consists of a parabolic dielectric slab with reflective coated edges where the light is coupled to it by two phase-shifted grating couplers and focused to a diffraction limited spot at the focal point of the parabola where a lollipop shaped NFT is located (Fig. 3)^10^,^12^,^25^. The reflection from the PSIM sidewalls causes the light to be coupled to the transducer with a broad spectrum of angles. However, to only excite a quadrupole mode one specific coupling angle is preferred as the other modes do not influence the field enhancement at the termination of the NFT. More precisely, the coupling angles different from the desired one only degrade the performance of the transducer through self-heating.

Unlike the PSIM coupling arrangement, the MZI-coupling design (Fig. 4)^26^,^27^ allows one to optimize the coupling efficiency to excite in the most efficient way only the resonance mode with the desired charge distribution^16^. Furthermore, the MZI coupling improves the control of the phase shift of the two beams coupled to the transducer. Appropriate selection of coupling angle and phase allows a matching to the radiation pattern of any antenna. In addition, it permits the coupling efficiency to the recording media to be maximized by integrating a transducer with a planar waveguide configuration where the power from laser to the waveguide can be coupled through the end-fire arrangement where coupling losses as low as 1–2 dB can be obtained.

The proposed Wheatstone bridge measurement configuration (Fig. 1) can be used to evaluate the efficiency of the coupling arrangement by measurement of the signal voltage for the same power from laser. Even excluding the coupling losses from the laser to the waveguide, the MZI–coupling arrangement ensures a much better coupling efficiency from the waveguide to the transducer. This enables a reduction in the power required from the laser to heat the recording media to a desired temperature. The Wheatstone bridge arrangement allows extraction of the power required to achieve a desired temperature rise by comparing the same signal voltage increase for different coupling arrangements. In addition, it allows an estimate of the temperature increase in the recording media through eq. 4.

#### Different transducer design

The NFT is the key component of the HAMR head as it enables breaking the diffraction limit and concentration of the optical energy into a spot which is not achievable by standard optical technology. It enables a large field enhancement within the optical spot that is required to deliver enough power to the recording media to change the material coercivity^10^. Apart from the field enhancement, the amount of power transferred to the recording media depends also on the impedance match between the heating media impedance (~1 kΩ) and the radiation resistance of the transducer^28^. This can be improved by increasing the transducer impedance which is achieved by increasing the transducer length.

**Figure 5:**
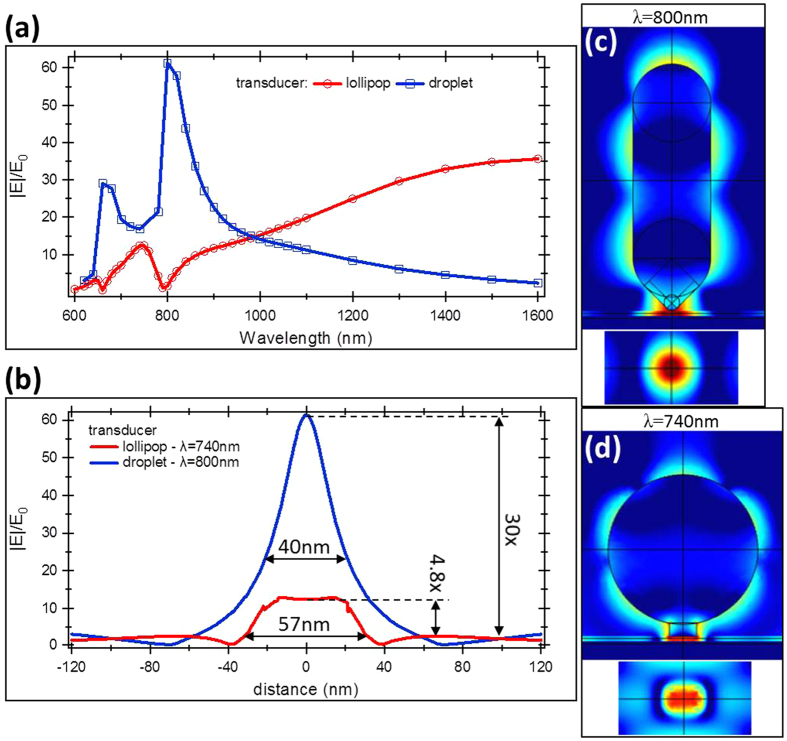
(**a**) Electric field enhancement of the lollipop and droplet transducers in close proximity (0.5 nm) to an image plane. (**b**) Electric field distribution along the width of transducers through the center at distance of a 7 nm from transducer and normalized to the E_0_. (**c**), (**d**) Electric field profile (logarithmic scale) through a cross-section of (**c**) droplet and (**d**) lollipop transducers.

The proposed Wheatstone bridge configuration can be a very convenient tool to evaluate the performance of the NFT in obtaining the temperature rise in the recording media. It can be implemented with any transducer design such as, for example, triangle antenna^29^, bowtie antenna^30^,^31^ or “nanobeak” design^32^,^33^. Here, we compared the field enhancement of two transducers, lollipop^23^,^34^ and droplet^15^, which can be easily integrated in a *2D* planar waveguide geometry either with the PSIM or MZI–coupling arrangement. The lollipop transducer parameters were chosen to be close to the state-of-the-art transducer design with a disk radius of *r* = 110 nm and peg dimensions of *w* = 40 nm and *l* = 20 nm being its width and length. For a droplet transducer design its width was kept at *w* = 100 nm and length at *l* = 320 nm. For both transducers the thickness was kept constant at *h* = 20 nm. As observed (Fig. 5) the field enhancement at the termination of the NFT is wavelength dependent with a maxima in the desired wavelength range of 700–900 nm, specifically at *λ* = 740 nm for the lollipop transducer and *λ* = 800 nm for the droplet transducer with corresponding field enhancements of ~12 and ~62 respectively. The higher field enhancement translates to a higher temperature rise in the recording media. It was previously estimated that the coupling efficiency of power transferred from the transducer to the recording media constitutes for only 5–7% of the total power coupled to the transducer^23^,^24^ and this is directly related to the large impedance mismatch between the recording media (~1 kΩ) and the lollipop transducer (~50–70 Ω). One way to increase the coupling efficiency is by increasing the transducer impedance which depends on the radiation resistance of the transducer which in turn depends on the length of the transducer. The droplet transducer design takes advantage of this point enabling the design of longer transducers with a higher coupling efficiency. The droplet transducer presented in this paper is about a 33% longer (*l* = 320 nm) compared to the lollipop transducer (*l* = 240 nm) which translates to a 33% increases in power transfer to the recording media. Together with a ~5 times higher field enhancement this leads to a ~7 times higher power transfer efficiency. However, compared to the field enhancement that can be easily estimated by using the SNOM measurement technique, the increased coupling efficiency related with the higher transducer length is difficult to measure. The Wheatstone bridge measurement configuration (Fig. 1) gives the possibility to estimate the overall power transfer efficiency and opens a route to efficient characterization of transducers in terms of power coupling efficiency and temperature rise of the recording media. With the help of this technique different plasmonic transducer designs can be easily evaluated.

As the SNOM measurement technique is an excellent tool for the evaluation of field enhancement and mode field confinement, the lack of measurement technique capable of estimating the temperature rise in the recording media poses a problem in the appraisal of different optical-NFT system for HAMR applications. Here, the Wheatstone bridge configuration was proposed to overcome those problems with a possibility to measure the temperature rise in the recording. Furthermore, it allows in easy way to evaluate both the efficiency of the transducer^35^ and the coupling arrangement by measuring a signal voltage drop for an applied bias voltage with very high accuracy. This new measurement technique opens the possibility for fast evaluation of different systems. It is, to our knowledge, the only technique capable of measuring the local temperature rise in a recording media which is of special interest for the data storage industry.

## Methods

The field enhancement calculations were performed using the three-dimensional finite element method (FEM) commercial software COMSOL Multiphysics with the dielectric constant of gold taken from literature^36^. For heat transfer calculations the thermal conductivity of the materials were taken as: FePt-*κ* = 5 (W/m·K)–lateral direction, and *κ* = 50 (W/m·K)–vertical direction^23^, SiO_2_-*κ* = 1.4 (W/m·K), Au-*κ* = 100 (W/m·K)^23^. A convection coefficient of 10 (W/m^2^·K) was assumed for a convective cooling of materials being in contact with air.

## Additional Information

**How to cite this article**: Gosciniak, J. *et al.* Wheatstone bridge configuration for evaluation of plasmonic energy transfer. *Sci. Rep.* **6**, 24423; doi: 10.1038/srep24423 (2016).
